# Eye orbit effects on eyeball resonant frequencies and acoustic tonometer measurements

**DOI:** 10.1038/s41598-022-08874-x

**Published:** 2022-03-22

**Authors:** Po-Jen Shih, Shao-Jie Wu, Ya-Hsing Sung, Yu-Ting Tung, Chia-Yu Chang, Shadie Hatamie, Zhi-Xuan Dai

**Affiliations:** 1 Department of Biomedical Engineering, National Taiwan University, No. 1, Sec. 4, Roosevelt Rd., Taipei, 10617 Taiwan, ROC; 2 Department of Mechanical Engineering, National Taiwan University, Taipei, Taiwan, ROC; 3 Department of Biomedical Engineering, National Cheng Kung University, Tainan, Taiwan, ROC

**Keywords:** Biomedical engineering, Acoustics

## Abstract

The eye orbit has mechanical and acoustic characteristics that determine resonant frequencies and amplify acoustic signals in certain frequency ranges. These characteristics also interfere with the acoustic amplitudes and frequencies of eyeball when measured with an acoustic tonometer. A model in which a porcine eyeball was embedded in ultrasonic conductive gel in the orbit of a model skull was used to simulate an in vivo environment, and the acoustic responses of eyeballs were detected. The triggering source was a low-power acoustic speaker contacting the occipital bone, and the detector was a high-resolution microphone with a dish detecting the acoustic signals without contacting the cornea. Dozens of ex vivo porcine eyeballs were tested at various intraocular pressure levels to detect their resonant frequencies and acoustic amplitudes in their power spectra. We confirmed that the eyeballs’ resonant frequencies were proportional to intraocular pressure, but interference from orbit effects decreased the amplitudes in these resonant frequency ranges. However, we observed that the frequency amplitudes of eyeballs were correlated with intraocular pressure in other frequency ranges. We investigated eye orbit effects and demonstrated how they interfere with the eyeball’s resonant frequencies and frequency amplitudes. These results are useful for developing advanced acoustic tonometer.

## Introduction

Intraocular pressure (IOP) is a key risk factor for glaucoma, and therefore, its measurement is critical for accurate diagnosis. IOP measurement with an air-puff tonometer assists the prognosis of patients with glaucoma and is widely used in clinics and hospitals^1^. However, access to IOP measurements outside hospital settings could further improve patient outcomes and convenience. Unlike the air-puff tonometer, in an acoustic tonometer, electro-acoustic technique is applied to measure IOP through frequency detection^2–4^. Because this tonometer consists of electro-acoustic components and does not require a large air chamber, it could be small, lightweight, and even integrated into consumer electronics such as smartphones. However, the resonant properties of the eyeball are affected by its environment and the surrounding bones. The resonant structure of the eye orbit can be analogized to that of a canyon, with the bones acting as the bedrock and the soft tissues as a sedimentary layer. Basin effects, as in earthquakes^5–7^, therefore cause interference in the duration, frequency, and amplitude of biomechanical responses of the eye. An incident wave into the tissues of the orbit is amplified by the overall resonant frequencies of tissues within the orbit. Although signals within the eyeball are simultaneously amplified, the orbit effects cause interference with both amplitudes and frequencies, and therefore, accurate measurement of these has remained out of reach.

Previous efforts toward analyzing the properties of the eyeball have focused on simulations^8^ or on mapping the measured resonant frequencies to corresponding IOP values^3,4^. The resonant frequency of the eyeball has a wide range, from 18 to 800 Hz (Table I in Shih et al.^9^). Note that the acoustic wavelength at the claimed resonant frequency is set in the scale over dozens of meters and is obviously much larger than the size of eyeball. The scattering waves are complicated, and thus, a simple mass-spring resonant model is not appropriate as well. The development of the fluid-filled and prestressed spherical model^9^ is based on the derivation of the Grinfeld’s soap bubble vibration theory^10^ (the fourth model in Table II) and the stretched plate vibration theory obtained from Mason’s book^11^. It has been proven that the sensitive range of this model is 20–140 Hz, which is also the range reported by Kim et al^2^. In acoustic tonometry, an acoustic trigger signal vibrates the eyeball, and then the frequencies or amplitudes of the responses from the cornea are detected. The trigger signal is generated by an acoustic speaker, which may be placed on the forehead^12^, temporal bone^13^, eyelid^14^, or set in front of the cornea^3^. The receiver, generally a microphone, makes contact with the eyelid or is placed in front of the cornea. However, all these triggering methods may produce eye orbit acoustic interference effects, limiting their effectiveness.

We considered orbit effects to design an acoustic tonometer in which the frequency ranges of the orbit and the resonant frequencies of the eyeball are accounted for. In our approach, a receiver with a dish was designed. The receiver can detect weak acoustic signals emitted from the cornea without making contact with it. The acoustic trigger is a speaker that generates low-energy signals from the occipital bone. The resonant frequencies of ex vivo porcine eyeballs were measured at various intraocular pressure levels. Eyeballs placed directly on the trigger and eyeballs embedded in an orbit were compared to identify their frequency ranges. We also tested conditions in which the receiver did or did not make contact with the cornea to compare the results and validate our approach. Our results from characterizing the major acoustic phenomena of the eyeball revealed a route toward developing an effective advanced tonometer.

## Results

**Figure 1:**
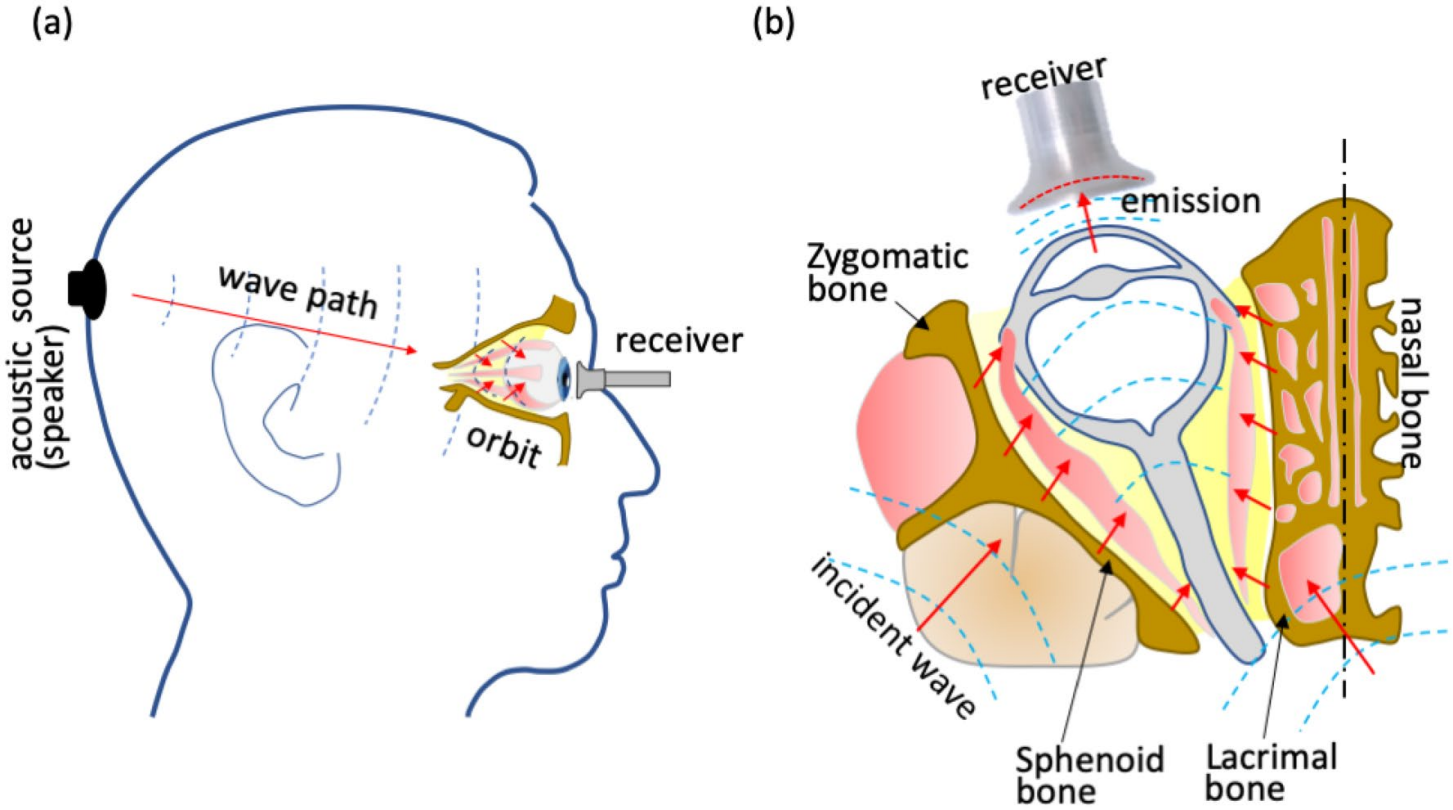
Illustration of the acoustic path and orbit effects. (**a**) Acoustic waves are emitted at the speaker and propagate to the orbit. (**b**) Some waves impinge on the orbit bones and then refract into muscle, fat, and the eyeball. Most of the refractive waves are trapped inside the orbit space. Finally, the eyeball surface emits waves into the air. These emission waves are detected by a receiver.

In our design, the acoustic wave is triggered at the occipital bone and propagates to the orbital bones. Then, the incident waves are emitted into orbital tissue. Because orbital bones are stiffer than orbital tissue, most waves refracting from bones are trapped inside the orbit (Fig. 1a). The cornea has a free surface, and it emits waves into air. Our dish-microphone receives these signals (Fig. 1b).

### Single-eyeball experiments

**Figure 2:**
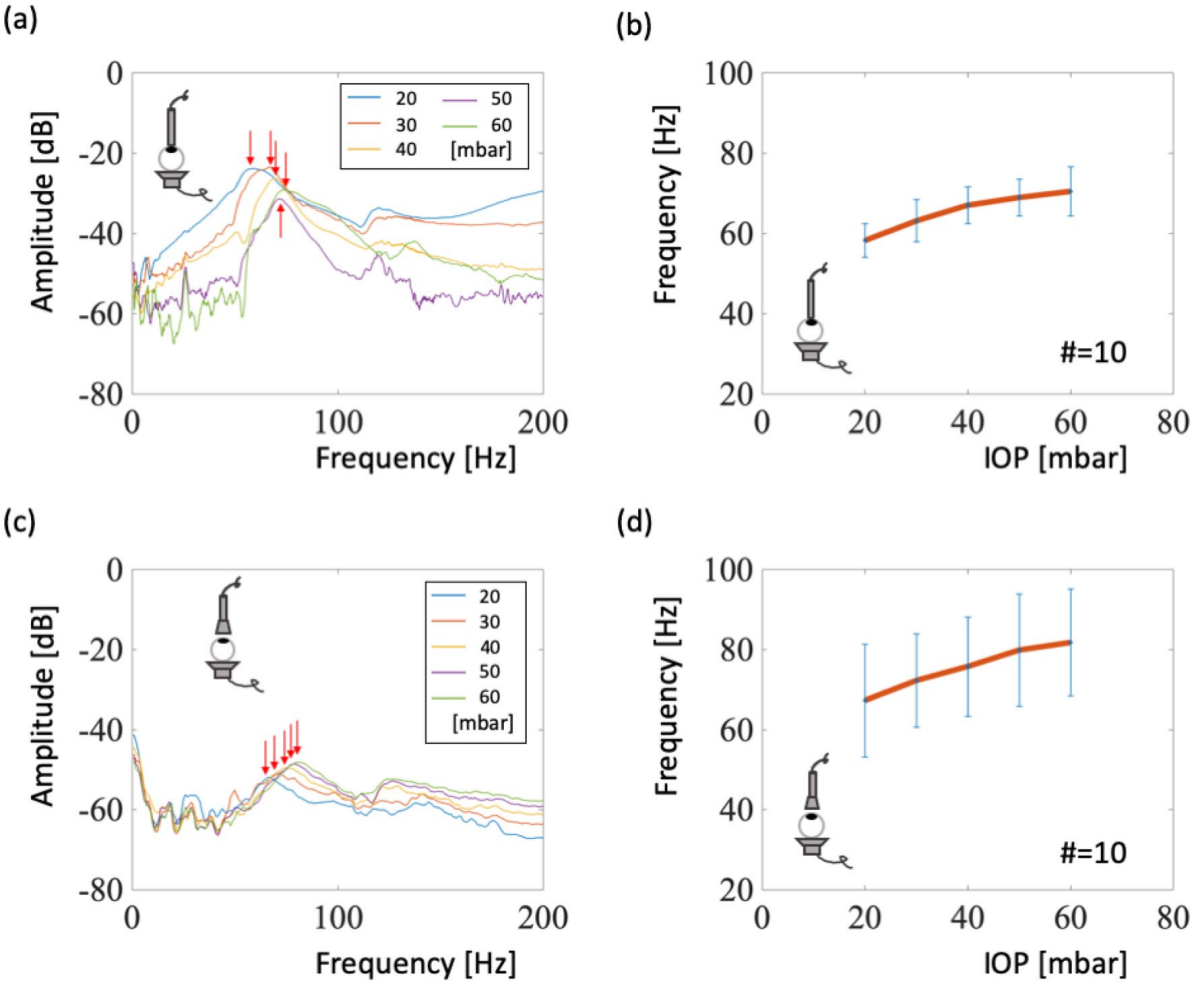
Contact and noncontact signals detected from an ex vivo eyeball. (**a**) Power spectra and frequency with an eyeball-contacting detector. Arrows shown the resonant frequencies at different IOP levels. (**b**) Statistical relationship between the resonant frequency and IOP for the contact experiment. (**c**) Power spectra and frequency with a noncontacting detector. (**d**) Statistical relationship between the resonant frequency and IOP for the noncontact experiment.

We designed contact and noncontact experiments to measure the resonant frequencies of the eyeballs at different IOP levels. For the single-eyeball contact experiment, we placed an eyeball on top of the speaker with the cornea upwards and with the microphone (without dish) contacting the top of the cornea. Figure 2a shows the power spectrum, indicating that resonant frequency increases with IOP. The amplitude–frequency relationship demonstrated that the resonant frequency shifted from 58 to 72 Hz as IOP increased from 20 to 60 mbar. The power spectra of tests on the other nine eyeballs are displayed in Fig. S1. These data also demonstrate a resonant frequency shift from 50 to 75.5 Hz among all eyes, as shown in Fig. 2b. Although the sizes and masses of the eyeballs are different, the resonant frequency shift from 20 to 60 mbar IOP was between 10 and 17 Hz for each eyeball. The average shift was 12.2 Hz, or 2.05 Hz per 10 mbar. In the noncontact experiments, the eyeball was placed on top of the speaker and the receiving dish was set 5 mm above the cornea, where the dish covered the whole cornea. The amplitude–frequency relationships demonstrated that the resonant frequency shifted from 67 to 81 Hz as IOP increased from 20 to 60 mbar, as shown in Fig. 2c. The results from experiments on the other nine eyeballs are shown in Fig. S2. The resonant frequency shift from 20 to 60 mbar IOP was between 7 and 26 Hz for each eyeball. The average shift was 14.5 Hz, or 2.42 Hz per 10 mbar.

### Orbit effects

**Figure 3:**
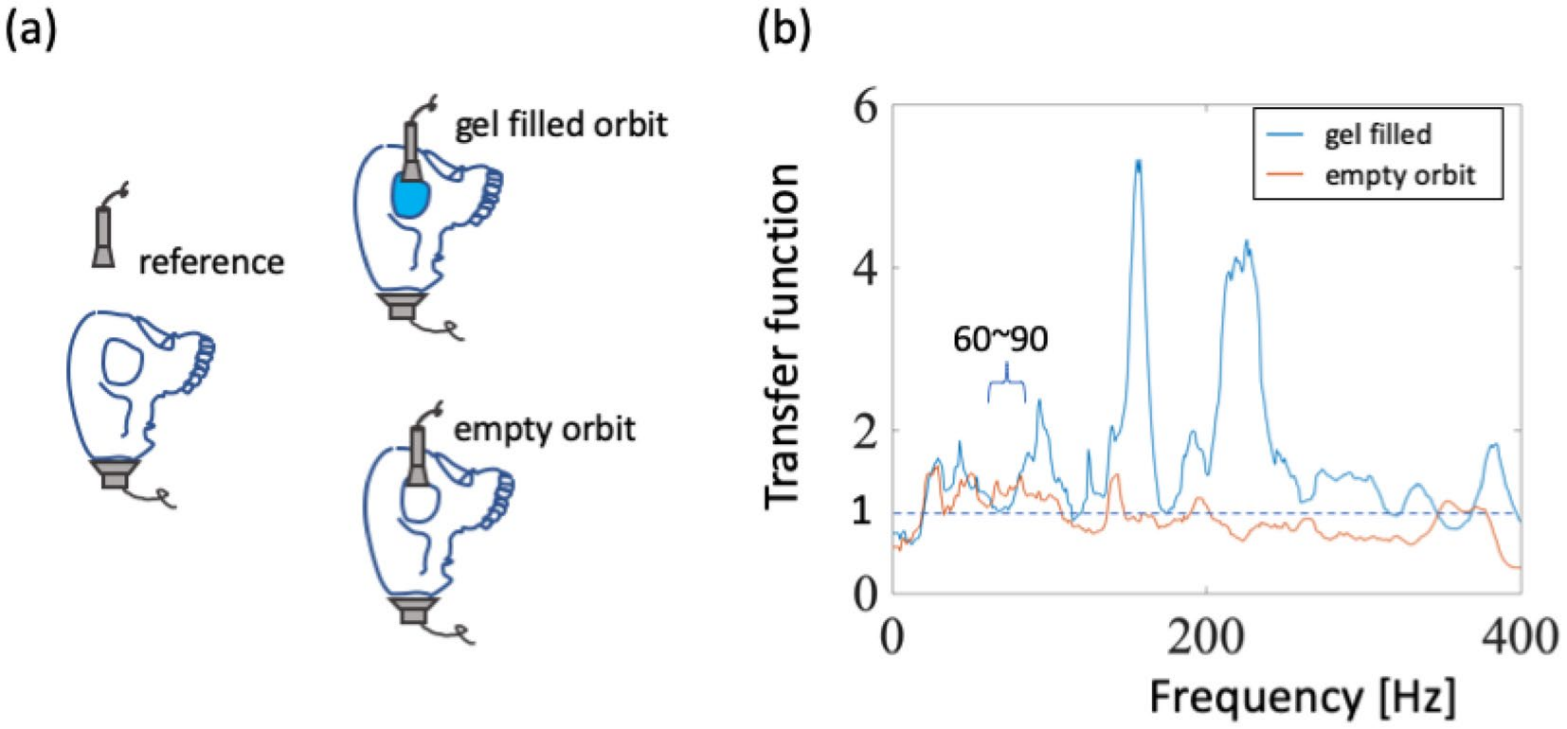
Orbital effects in relation to a reference signal. (**a**) Illustration of different receiver positions and orbit conditions. (**b**) Transfer functions in the frequency domain in relation to the reference signal.

To study the acoustic properties of the orbit structure, we used a model skull to simulate the human head, and we filled the orbit with ultrasound conductive gel to simulate orbital tissue. First, a ground reference signal was measured by triggering the speaker at the occipital bone, emitting acoustic waves received by the dish microphone placed approximately 3 cm in front of the forehead. Then, signals from an empty orbit and signals from a gel-filled orbit were measured by the dish microphone placed above the orbit approximately 1 cm (Fig. 3a). The transfer functions of these two signals were obtained by comparing them with the ground reference signal. In Fig. 3b, the frequency spectrum shows that the gel enhances amplitudes in several frequency ranges, especially at 70–250 Hz. In particular, the frequency range of signals from the eyeball, 70–100 Hz (Fig. 2), is also amplified by the gel. Therefore, filling the orbit with gel caused the frequency response to be more similar to that of an actual eyeball compared with in the empty orbit experiment, demonstrating that a gel-filled orbit it is an appropriate substitute for orbital tissue.

### Eyeball embedded in skull experiments

**Figure 4:**
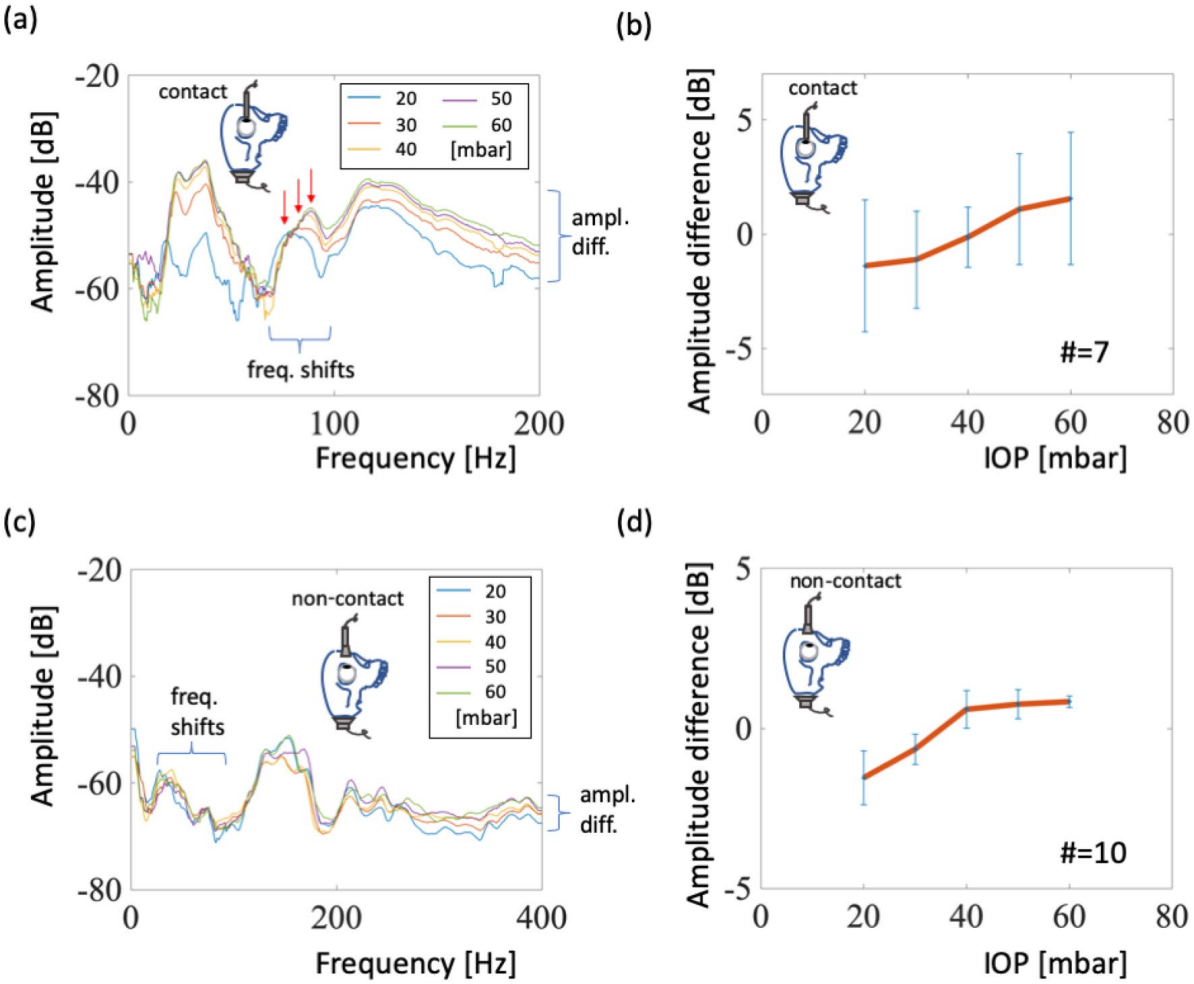
Contact and noncontact signals from an embedded eyeball. (**a**) Power spectra for the contacting eyeball test. Arrows indicate the resonant frequencies of various IOP levels. (**b**) Statistical results relating amplitude differences and IOP levels. (**c**) Power spectra for the noncontact eyeball test. (**d**) Statistical model relating amplitude differences and IOP levels.

We explore this setup by embedding a porcine eyeball into the gel-filled orbit. The gel simulates muscles and fats in the orbit and helps transfer acoustic signals from bones to eyeballs. Contact and noncontact experiments were performed to investigate this the reliability of the setup. Figure 4a displays the best result from several contact tests; the power spectrum shows a frequency shift from 79 to 89 Hz as IOP increases from 20 to 60 mbar. Although similar frequency shifts were observed in our other tests, these shifts were less clear (Fig. S3). On the other hand, we attempted a different approach instead of measuring frequency shifts. We measured the average amplitude of signals between 135 and 165 Hz. The increases in average amplitudes, as shown in Fig. 4b, indicate that as IOP increased from 20 to 60 mbar, the average amplitude increased by 3 dB. In the noncontact tests, the experiment was similar, but the dish microphone was placed 5 mm above the cornea. The results are shown in Fig. 4c. The frequency shifts can also be observed in a certain frequency range from 50 to 90 Hz. Furthermore, the response amplitudes at different IOP levels were in the range of 200 to 400 Hz, a pattern observed in the other nine tests (Fig. S4). Therefore, these average-amplitude increased by 3 dB as IOP increased from 20 to 60 mbar, as shown in Fig. 4d.

## Discussion

The orbit and orbital tissues amplified responses from 150–160 Hz input frequencies up to 5.3 times (Fig. 3b); but at 60–90 Hz, the frequency range relevant for IOP measurement (Fig. 2), amplification was relatively weak. On the other hand, results from other researches in basin acoustics are applicable to this problem; for waves in an empty and triangular canyon, the displacement transfer function was revealed to be 0.5–1.5 within the canyon^15^. Another study in which waves near a dike were modeled demonstrated that the transfer function value to be approximately 10 at the top of the dike^16^. This value was larger than in our experiment (5.3) because the larger value was calculated near the resonant frequency. Additionally, when the actual composition of the eye orbit is considered, including bone, muscle, fat, and the eyeball, it is likely that larger relative amplitudes will be measured because bone is stiffer than our plastic model. Moreover, the resonant frequency range is determined by the ratio between the geometric diameter of the orbit and the incident wavelength^17^. In our experiment, the incident wavelengths were hundreds of times larger than the orbit’s geometric diameter. Therefore, the duration of the incident waves, a parameter used to calculate basin effects, was not measured because the orbital diameter was too small compared with the acoustic wavelengths.

The resonant frequency shift of an eyeball depends on the tension strength of the membrane, which contains the cornea and sclera, when an increase in internal pressure expands and stretches the membrane^9^. As the membrane stretches, its stiffness increases^18^. A stiff membrane permits higher wave velocities, and that results in higher resonant frequency of the membrane. Despite differences in an eyeball’s size and weight, the resonant frequency range of a single eyeball is stable and repeatable at 60–90 Hz (Fig. 2), better than wide ranges presented in other studies (references are listed in Table I of Shih et al.^9^). The resonant frequency shift of an eyeball was 12–14 Hz when pressure was increased from 20 to 60 mbar. For eyeballs embedded in model skull orbits, we measured the amplitude differences between certain frequency ranges (135 and 165 Hz) to replace resonant frequency shift detection because of orbital characteristics influencing the resonant frequency range of the eyeball. Our result also indicates that the resonant frequency range of a gel-embedded eyeball (75–100 Hz, Fig. 4a) is higher than that of a single eyeball (60–80 Hz, Fig. 2a). Because the gel surrounding the soft eyeball supports it, resulting in a stiffer membrane during oscillation and high resonant frequencies. The amplitude differences are small and would require individual calibration if this tonometer were to be commercially produced.

Amplitudes^19^, amplitude changes^3^ in the time domain, or frequency shifts^20^ in the frequency domain could be measured using this technique. The change in the magnitude of the vibration response, based on the root mean square acceleration over time, could also be correlated with IOP^2^. Considering this result, we studied the frequency shift^9^ and expanded on this research to measure the amplitude changes in the frequency domain. From our experiments, we observed that the amplitudes across the frequency spectrum were sensitive to the contact force applied on the cornea. Details could observe the variation of the maximum amplitudes near the resonant frequencies in every test of Fig. S1. Therefore, the noncontact method, which avoids corneal contact, is a substantial advancement in acoustic tonometer development. The key factor is the dish design to assist the microphone to collect acoustic energy from the cornea to the microphone. Therefore, the noncontact technique helps patients operate this tonometer at home without bacterial contamination or risk of corneal injury.

**Figure 5:**
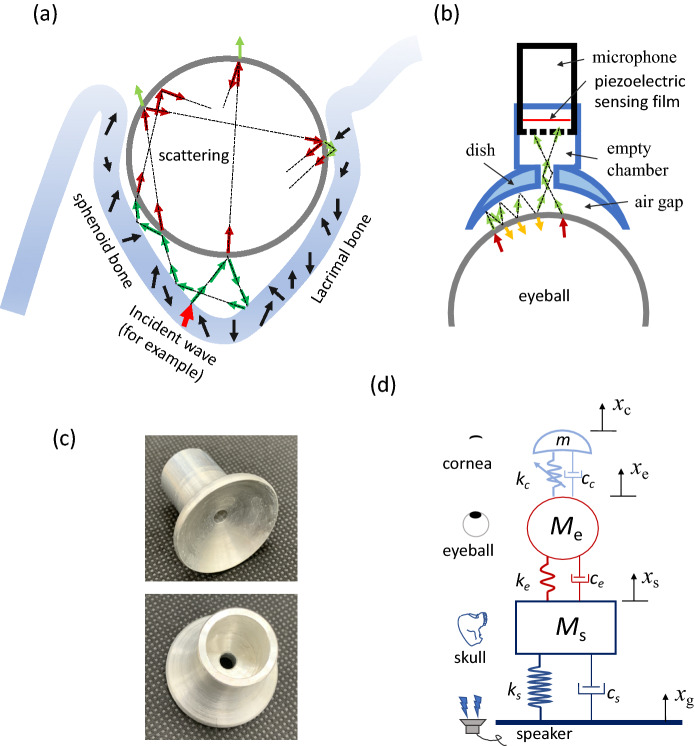
Wave propagation and modeling. (**a**) One wave in skull (considering the red arrow, for example) propagates into the tissues and then into the eyeball. It is reflected, refracted, and scattered many times and is transformed into many waves. All these waves are trapped by the bone boundaries, but not the corneal boundary. (**b**) The waves emitted from the cornea are trapped again by the dish boundary, but not by the central hole of the dish. The waves were reflected several times and then propagated into the empty chamber. (**c**) Two photos of the dish. A small hole is connected to the empty chamber. (**d**) The simplified mass-spring-damper model. The equivalent springs, the equivalent dampers, and the equivalent masses describe the eyeball embedded in the skull and the intraocular pressure is modeled by adjusting the corneal spring constant.

The quality of experimental results of the eyeball and the skull mainly relies on the resonance characteristics of acoustic materials, interfaces, and structures. In theory, the acoustic signals entering the eyeball from the skull interfere with each other after being reflected multiple times by the hard boundaries of the skull, enhancing the signal intensity in a certain frequency range, like an acoustic prison structure^21,22^, as shown in Fig. 5a. When external energy is transmitted stably from the skull, the eyeball is equivalent to an acoustic source, and energy can only be emitted outward from the cornea. Unfortunately, there are many tissues in the eyeball, such as the lens, iris, and vitreous; these enable energy absorption and protect the eyeball’s safety. These tissues lead to wave dissipation, and the signals become weak. To circumvent this issue, the authors also designed another acoustic prison in the air gap between the cornea and the dish microphone, as shown in Fig. 5b. The radius of the dish curvature is slightly smaller than that of the cornea to create a prison space. The acoustic signals are emitted into the air gap from the cornea and are reflected multiple times by the hard dish and cornea, and the boundary shapes cause the waves to be propagated into the central hole of the dish. The hole size contributes to improving the localized effects on the amplitude signals and enhancing the resolution, as shown in Fig. 5c. Therefore, the hole side range was adjusted to fit the frequency range in this eyeball experiment. Behind the small hole, we also designed a cavity chamber, which is another acoustic prison, to reshape the signal; thus, acoustic pressure can be applied onto the piezoelectric sensing film of the microphone. In this paper, we have provided the bandwidths for designing an acoustic tonometer, and accordingly, a customized microphone could be developed in the future^23^.

To simplify the waves in the eye orbit effect, we employed the spring-mass model to explain the physical image (demonstrated in Fig. 5d). The speaker represents the vertical input with a displacement, denoted by *x*_g_. The mass, stiffness, and viscosity are simulated by the equivalent masses (*M*_s_, *M*_e_, and *m*), the equivalent spring constants are denoted by (*K*_s_, *K*_e_, and *K*_c_), and the equivalent damping coefficients are denoted by (*c*_s_, *c*_e_, and *c*_c_) for the skull, eyeball, and cornea, respectively. Further, the equivalent displacements for the equivalent masses are denoted by *x*_s_, *x*_e_, and *x*_c_ for the skull, eyeball, and cornea, respectively. Since the skull is much stiffer than the eyeball, the *K*_s_ is larger than the *K*_e_, and this stiffness mismatch leads to a low transition from the skull to the eyeball. The soft spring, *K*_e_, enlarges the amplitude response of the eyeball, i.e., *x*_e_ > *x*_s_, and transmits less signal back to the skull. In addition, the spring constant in the cornea is adjustable and is dependent on the intraocular pressure. Thus, the frequency and amplitude signals detected from the corneal surface contain characteristics of the intraocular pressures.

The boundary constraints between the eyeball and the skull may affect the refractive and reflective waves, especially, the frequencies dominated by the geometry and material properties of the soft matter^24^. In anatomy, the space between the eyeball and the skull is a conical structure and contains the muscles, the optic nerve, and fat. In our experiment, we utilized a conductive gel to fill the cone space and simulate these tissues. The volume of the used gel is about 2/3rd the volume of the eyeball. This conductive gel is commonly used in both diagnostic and therapeutic ultrasounds. The ingredients of the conductive gel are carbomer polymer, propylene glycol, glycerol, and methylparaben, and the sound speed of the gel is in the range of 1460–1650 m/s (for humans)^25^. Even the conductive gel affects the shifts of the real frequencies of the orbit; the functions of the conductive gel are to transmit energy from the skull to the eyeball and to absorb energy through the orbit effect. The power spectrum of the gel-filled orbit is shown in Fig. 3.

For the experimental design, we chose the commonly used microphone, instead of the high-accuracy laser device. The laser Doppler vibrometer has been utilized to measure the intraocular pressure ex vivo^26^. The He:Ne laser (633 nm) is categorized as a class-two laser and is safe for momentary exposures; however, deliberately staring into the beam from this laser is hazardous. Even though the acoustic signals are weak, the authors designed a special dish and covered it on the top of the microphone to increase the amplitude. The transmission of the power spectrum is shown in Figs. S5, S6, and S7 and the corresponding details are provided in the Supplementary material. On the other hand, the acoustic waves triggered in the low-frequency range (< 400 Hz) are safe, and they can even be produced by human vocal cords, as the wave sources for in vivo tests in the future.

The limitations of this study primarily relate to the porcine eyeball and the skull model. First, even though the diameters of porcine and human eyeballs are similar, the frequency responses obtained from porcine eyeballs cannot exactly simulate those obtained from human eyeballs. Second, the skull model is plastic and less stiff than real bone, refracting less waves from plastic orbit bones into tissue, and it results in less waves can be detected from cornea. Third, the skullcap model was not considered in this study because it was separated from the skull in the educational model. Thus, the frequency spectra in our study do not include the contributions from the skullcap or other orbital tissues.

Our investigation provided three findings. First, we describe the effects of orbital tissue and geometry on orbit acoustics and revealed amplification in a certain frequency range. Signal interference within the orbit was caused by certain eyeball characteristics. Second, the mechanical properties of an eyeball embedded in an orbit are different from those of an eyeball removed from the orbit. Therefore, it is necessary to consider the entire eye-orbit system instead of single eyeballs in future research. Third, the detection of amplitude changes is inappropriate for contact detection because amplitudes are sensitive to contact conditions. Moreover, noncontact methods can be effective when the dish is applied to enhance signal. Our study had achievements in the detection of eyeball signals and clarified some difficulties in acoustic tonometer development.

## Methods

### Sample preparation for frequency tests

The fresh porcine eyeballs were collected from Cheng-gong and Shuiyuan Markets (Taipei, Taiwan), and 2–4 eyeballs were tested per day. The eyeballs were moistened to maintain hydration. Frozen eyeballs or eyeballs with opaque corneas were not used in our tests. The results of tests on 37 eyeballs are presented in this study; hundreds of eyeballs were tested in the design of the experimental process and development of the methodology. The six extraocular muscles and surrounding fats were removed, and the optic nerve was trimmed flush to the sclera. In the skull test, an eyeball was embedded into the orbital cavity and was surrounded by ultrasound conductive gel so that the eyeball did not contact the model skull. As this study used only animal parts obtained from markets, the requirement to obtain approval from the institutional animal care and committee was waived.

### Equipment and setup

**Figure 6:**
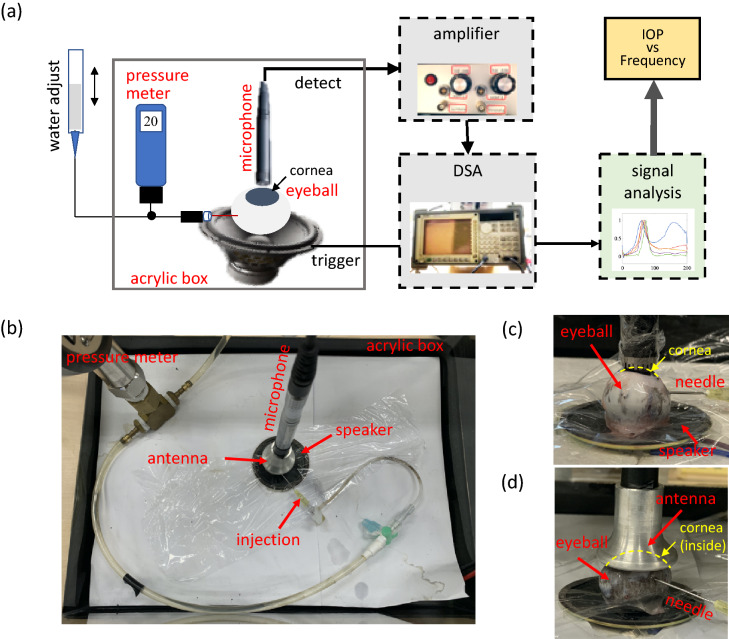
(**a**) Schematic of the equipment setup. (**b**) Experimental setup in the acrylic box (top view). (**c**) Receiver gently contacting the eyeball. (**d**) Dish covering the eyeball without contact.

To measure the resonant frequency of the eyeball at different IOP levels, a periodic-sweep-signal source was applied to the speaker. The experiment was conducted in an acrylic box of size 35 cm × 26 cm × 32 cm. A piece of cardboard covered the top of the box, and Styrofoam as well as a plastic cushion were used as a sound barrier below the box. Three systems were used in the experiment: the eye pressure adjustment system, the vibration triggering system, and the signal processing system. A schematic of system setup is shown in Fig. 6a. For the eye pressure adjustment system, a water tank with a pipe was connected in parallel to a pressure gauge and connected to a 27-gauge syringe needle in series. The needle injected water into the vitreous body of the porcine eyeballs. According to the principle of communicating vessels, the IOP of an eyeball would change with tank water level; IOP could be set at 10-mbar intervals from 20 to 60 mbar. For the acoustic system, a speaker (DS, YD 58-3R, 8 Ω and 0.5 W) vibrated the eyeball from below.

**Figure 7:**
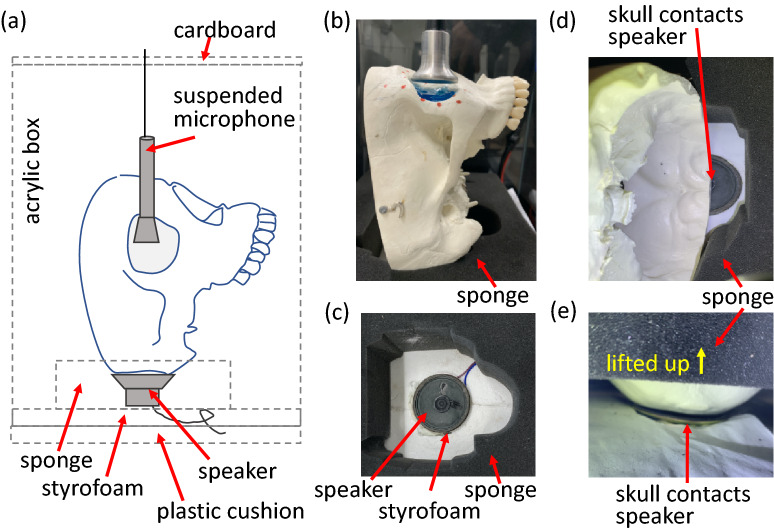
(**a**) Schematic of the equipment setup. (**b**) Skull setup including the gel-filled orbit, the supporting sponge, and the noncontact receiver. (**c**) Speaker arrangement. (**d**,**e**) The skull contacts the speaker with its curved surface.

**Figure 8:**
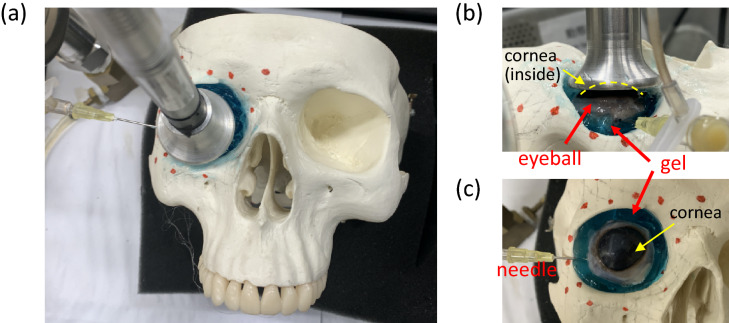
(**a**) Experimental setup in the acrylic box (top view). (**b**) Eyeball embedded in a gel-filled orbit and receiver setup. (**c**) Syringe needle setup.

The eyeball was placed with the cornea upward facing the microphone. A high-resolution microphone (Brüel and Kjær, 4189) was used to detect acoustic signals. A Keynet 35670A Dynamic Signal Analyzer (DSA, Agilent) applied a periodic sweep-signal from 0 to 200 Hz with 0.5 Hz resolution and a triggering voltage from − 1 to + 1 to the speaker (for the single-eyeball experiment, as shown in Fig. 6b–d). For the orbit test (Figs. 7, 8), the frequency range was 0–400 Hz with a − 3 to + 3 triggering voltage. The output power from the speaker is 1.125 W. The DSA also received signals from the microphone and converted them into power spectra.

### Experimental procedures for measuring power spectra

We varied the IOP of the eyeball and measured associated power spectra to determine each eyeball’s frequency properties. To measure the resonant frequency region, the microphone was placed in gentle contact with the cornea and detected signals from its surface, as shown in Fig. 6c. Ten porcine eyeballs were measured at five pressure levels (20, 30, 40, 50, and 60 mbar). Ten tests were conducted at each pressure level, and the results were averaged. To avoid interference from the contact surface, we also performed a noncontact experiment, with the dish microphone placed 5 mm above the cornea, as shown in Fig. 6d. As in the contact experiment, resonant frequencies were measured 10 times at five pressure levels, and the results were averaged.

To explore the effects of orbital bone and tissue in the frequency domain, we obtained baseline results from a model skull. We filled the orbit of the model skull with ultrasound conductive gel, as shown in Fig. 7a. The skull was oriented vertically to stabilize the gel and avoid gel outflow. The microphone with a dish was placed 1 cm above the gel, as shown in Fig. 7b. The bottom of the skull model was in contact with the speaker, and the bottom part of the skull was supported by a sponge (Fig. 7c).

Finally, we embedded an eyeball into the gel-filled orbit model to simulate the environment of a human orbit. Frequency response testing was performed using two setups, with the microphone contacting the cornea and placed 5 mm above the cornea. In the noncontact type, the dish was placed carefully to avoid contacting the bones or eyeball (Fig. 8a, b), and the syringe needle was placed without contacting the bone (Fig. 8c). The frequency response of the porcine eyeballs was measured at five pressure levels from 20 to 60 mbar, and the results of 10 tests at each pressure level were averaged.

### Signal analysis and transfer functions

The acoustic response in the time domain is automatically converted into a response in the frequency domain by the DSA. Thus, the DSA displays the power spectrum^27^ directly. The power spectrum displays the received signal in relation to the emitted frequency. In Fig. 3b, the transfer function, $T\left(f\right)$, is defined as the ratio between two power spectra, $T(f)=X(f)/G(f)$, where $X(f)$ is the power spectrum of the eyeball and $G(f)$ is the power spectrum of the ground signal. In Fig. 4, the amplitude difference over a certain frequency range is defined as $\Delta X\left(f\right)=X\left(f\right)-\overline{X }(f)$, where $X(f)$ is the average amplitude of the power spectrum at a certain IOP, and $\overline{X }(f)$ is the average amplitude of all power spectra across all IOP levels.

## Supplementary Information

Supplementary Information.

## References

[1] Forbes M, Pico G, Grolman B (1974). A noncontact applanation tonometer: Description and clinical evaluation. Arch. Ophthalmol..

[2] Kim D (2021). A pilot study for intraocular pressure measurements based on vibroacoustic parameters. Sci. Rep..

[3] von Freyberg A (2009). Acoustic tonometry: Feasibility study of a new principle of intraocular pressure measurement. J. Glaucoma.

[4] Krakau CET (1970). A vibration tonometer. Ophthalmic Res..

[5] Somerville, P., Collins, N., Graves, R. & Pitarka, A. in *Proceedings of the 13th World Conference on Earthquake Engineering*.

[6] Ayoubi, P., Asimaki, D. & Mohammadi, K. Basin effects in strong ground motion: A case study from the 2015 Gorkha, Nepal earthquake. arXiv preprint arXiv:1807.00950 (2018).

[7] Rial JA, Saltzman NG, Ling H (1992). Earthquake-induced resonance in sedimentary basins. Am. Sci..

[8] Keiper DA, Sarin LK, Leopold IH (1965). The vibration tonometer. I. Principles and design. Am. J. Ophthalmol..

[9] Shih P-J, Guo Y-R (2016). Resonance frequency of fluid-filled and prestressed spherical shell—A model of the human eyeball. J. Acoust. Soc. Am..

[10] Grinfeld P (2012). Small oscillations of a soap bubble. Stud. Appl. Math..

[11] Mason WP (1942). Electromechanical Transducers and Wave Filters.

[12] Lenhardt, M. M. & Ward, K. Method and apparatus for monitoring intra ocular and intra cranial pressure. U.S. Patent and Trademark Office US patent no. 8,172,769 B2 (2004).

[13] Shih, W. P., Shih, P. J., Yen, J. Y. & Wang, I. J. Acoustic wave intraocular pressure detecting device and method thereof. US Patent and Trademark Office US patent no. 10194886 B2 (2014).

[14] Uchiyama, A., Yanashima, K., Takeda, S., Taguchi, G. & Yokoyama, T. Method and device for measuring intraocular tension. National Intellectual Property Administration CN patent no. 1511009A (2002).

[15] Shyu WS, Teng TJ, Chou CS (2018). Effect of geometry on in-plane responses of a symmetric canyon subjected by P waves. Soil Dyn. Earthq. Eng..

[16] Shyu WS, Teng TJ, Chou CS (2017). Anti-plane response caused by interactions between a dike and the surrounding soil. Soil Dyn. Earthq. Eng..

[17] Yeh CS, Teng TJ, Shih PJ (2004). On formulation of a transition matrix for poroelastic medium and application to analysis of scattering problem. J. Acoust. Soc. Am..

[18] Boschetti F, Triacca V, Spinelli L, Pandolfi A (2012). Mechanical characterization of porcine corneas. J Biomech Eng.

[19] Dubois P (2007). A new method for intra ocular pressure in vivo measurement: First clinical trials. Annu. Int. Conf. IEEE Eng. Med. Biol. Soc..

[20] Alam SK, Richards DW, Parker KJ (1994). Detection of intraocular-pressure change in the eye using sonoelastic doppler ultrasound. Ultrasound Med. Biol..

[21] Ma FY, Chen JY, Wu JH, Jia H (2020). Realizing broadband sub-wavelength focusing and a high intensity enhancement with a space-time synergetic modulated acoustic prison. J. Mater. Chem. C.

[22] Ma FY, Chen JY, Wu JH (2019). Three-dimensional acoustic sub-diffraction focusing by coiled metamaterials with strong absorption. J. Mater. Chem. C.

[23] Ma FY, Wang C, Liu CR, Wu JH (2021). Structural designs, principles, and applications of thin-walled membrane and plate-type acoustic/elastic metamaterials. J. Appl. Phys..

[24] Ma FY, Wang C, Du Y, Zhu ZC, Wu JH (2021). Enhancing of broadband sound absorption through soft matter. Mater. Horiz..

[25] Othman NS, Jaafar MS, Rahman AA, Othman ES, Rozlan AA (2011). Ultrasound speed of polymer gel mimicked human soft tissue within three weeks. Int. J. Biosci. Biochem. Bioinform..

[26] Salmi A, Nieminen HJ, Veira Canle D, Haeggstrom E, Kontiola A (2020). Non-contact determination of intra-ocular pressure in an ex vivo porcine model. PLoS ONE.

[27] Semmlow J (2012). Signals and Systems for Bioengineers.

