# Supplementary material for: Eye orbit effects on eyeball resonant frequencies and acoustic tonometer measurements
Source: Sci Rep. 2022 Mar 22;12:4883. doi: 10.1038/s41598-022-08874-x (PMC8941096; doi:10.1038/s41598-022-08874-x)
Supplement: Supplementary file 1 — Supplementary Information. [file 41598_2022_8874_MOESM1_ESM.docx]

**Eye orbit effects on eyeball resonant frequencies and acoustic tonometer measurements**

Po-Jen Shih^1*^, Shao-Jie Wu^2^, Ya-Hsing Sung^1^, Yu-Ting Tung^3^, Chia-Yu Chang^2^, Shadie Hatamie^1^, and Zhi-Xuan Dai^2^

^1^Department of Biomedical Engineering, National Taiwan University, Taiwan

^2^Department of Mechanical Engineering, National Taiwan University, Taiwan

^3^Department of Biomedical Engineering, National Cheng Kung University, Taiwan

Correspondence and requests for materials should be addressed to Po-Jen
Shih (pjshih@ntu.edu.tw)

**Supplementary text**

**Frequency responses of acoustic speaker**

For the microphone, the free-field frequency response of the microphone is around 0 dB in the frequency range from 20 to 1000 Hz, as shown in Figure S5(a). In addition, the frequency response between the speaker and the microphone was tested via the contact and noncontact tests (as shown in Figure S5(b)). Note that when the microphone contacted the central surface of the speaker since too close, it was covered by a membrane on the dish top (like a stethoscope); the dish without the membrane was utilized for detecting acoustic signals in the air gap between the microphone and the speaker. The membrane was a tensed plastic wrap used to create an air chamber inside the curve disc. The frequency responses of the contact and noncontact tests are shown in Figure S5(c). The test performed under the contact condition showed that the frequency response was around -10 dB, but the radiation in the air gap reduces large amplitudes at such low frequency ranges.

**Frequency responses of skull**

The speaker was set to contact the occipital bone, and the loudspeaker transmitted vibration signals into the skull. In the experimental design, the speaker represented a vibrator and was supported by solid ground. On the other hand, the skull was supported by a soft sponge that helped reduce environmental interactions with the boundaries. The sponge represented the spring required to support the skull. The speaker acted like a tapping source, hitting the skull with specific signals. The tapping source triggered the acoustic waves to travel and scatter in the skull. Therefore, slight vibrations on the skull surface could be felt during the experiment. In addition, we measured the frequency responses with the dish microphone placed at the forehead surface (approximately 3 cm in front of the forehead), as shown in Figure S6. Note that the dish microphone covered with a membrane was only used to contact the skull surface, and the ultrasound conductive gel was placed in between to fill the irregular surface. The frequency response showed that the amplitude of the acoustic waves in the skull was about -20 dB to -30 dB, relative to the low amplitude of the acoustic waves measured in the air.

**Transmission and attenuation**

The results from Figures 4, S5, and S6 were compiled to investigate the transmission and attenuation between the mediums and boundaries. As shown in Figure S7, the results obtained from five different setups were compared: (1) the dish microphone contacted the speaker (from the blue curve in Figure S5(c)). (2) The dish microphone contacted the forehead of the skull, which represented the transmission from the speaker to the skull (from the red curve in Figure S6(b)). (3) The microphone contacted the eyeball, and the eyeball was embedded in the skull; this represented the transmission from the speaker, through the skull, to the eyeball (from the 30-mbar curve in Figure 4(a)). Note that in this contact test, the frequency shifts were considered in the range of 70–100 Hz. (4) The dish microphone did not show contact with the embedded eyeball. This represented the acoustic signals that were emitted from the cornea and detected by the dish microphone (from the 30-mbar curve in Figure 4(c)). Note that under the non-contact conditions, the amplitudes were considered in the range of 200–400 Hz. (5) The dish microphone was placed in front of the forehead with a 1-cm air gap, which represented the ground reference signals (defined in Figure 3 and by the orange curve in Figure S6(b)). In conclusion, the diffracted signals in the air were weaker than those in the skull. Therefore, to differentiate between the intraocular pressures, we measured the frequency shifts in the contact tests and the frequency amplitudes in the non-contact tests.

**Supplemental figures**

**
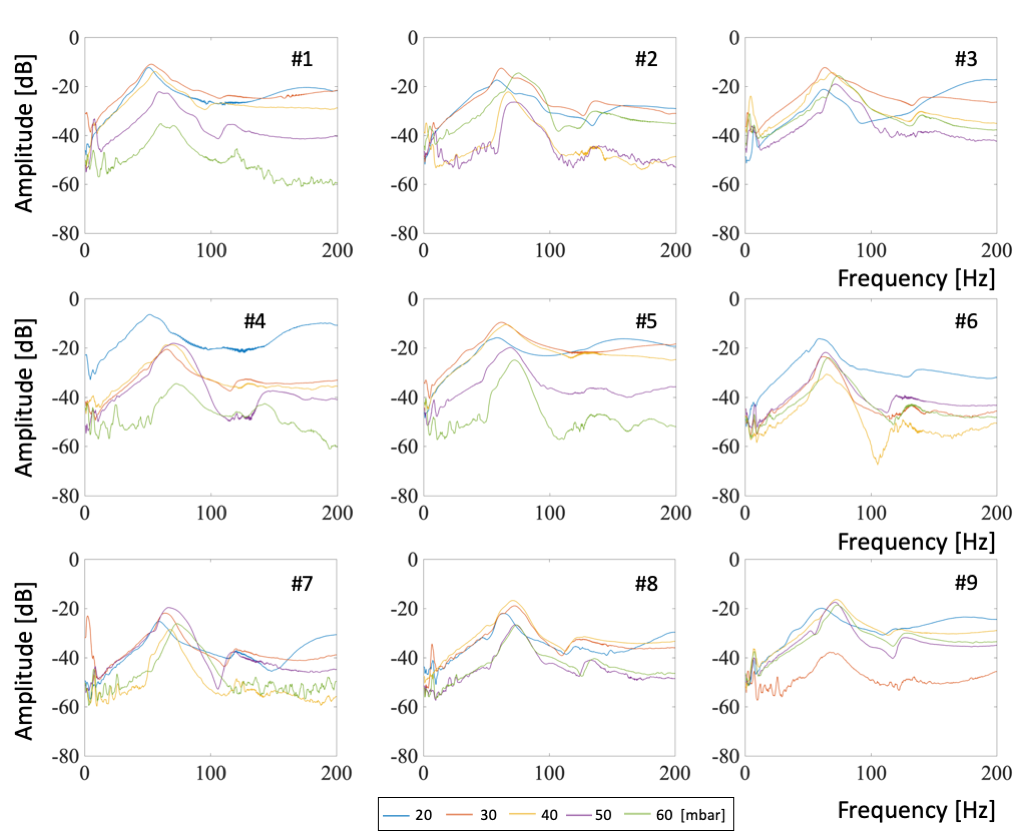
**

Figure S1 Nine power spectra from the single-eyeball contact tests


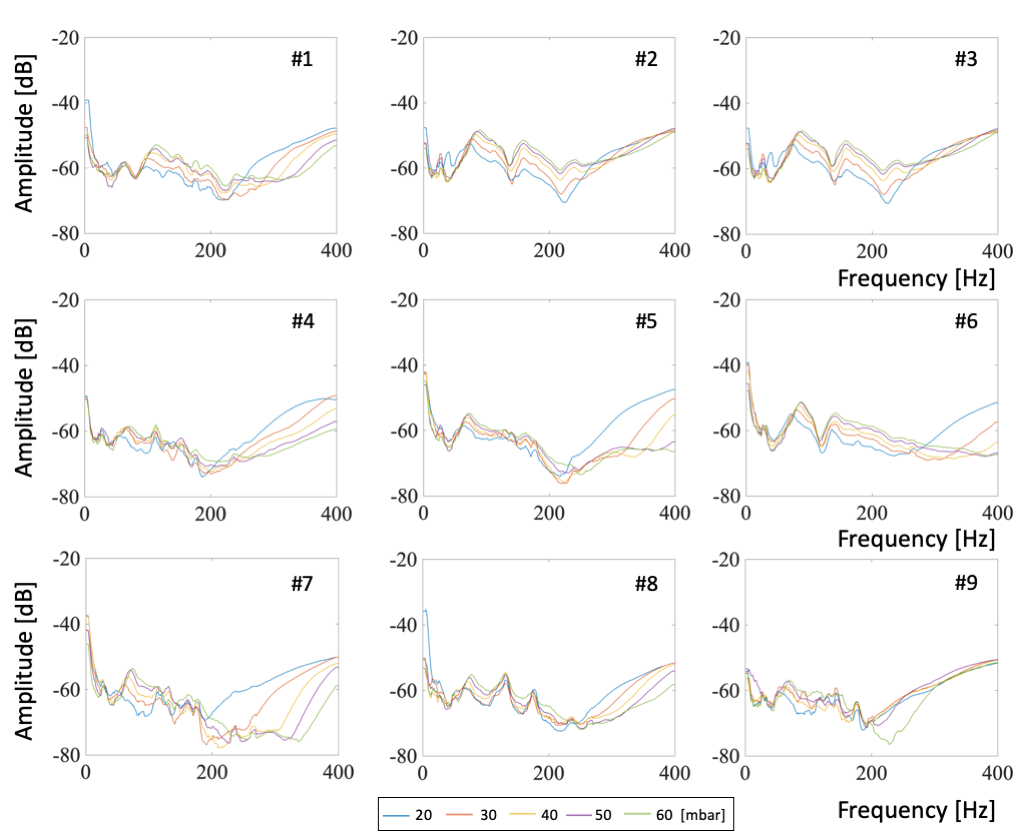


Figure S2 Nine power spectra from the ex vivo eyeball noncontact tests.


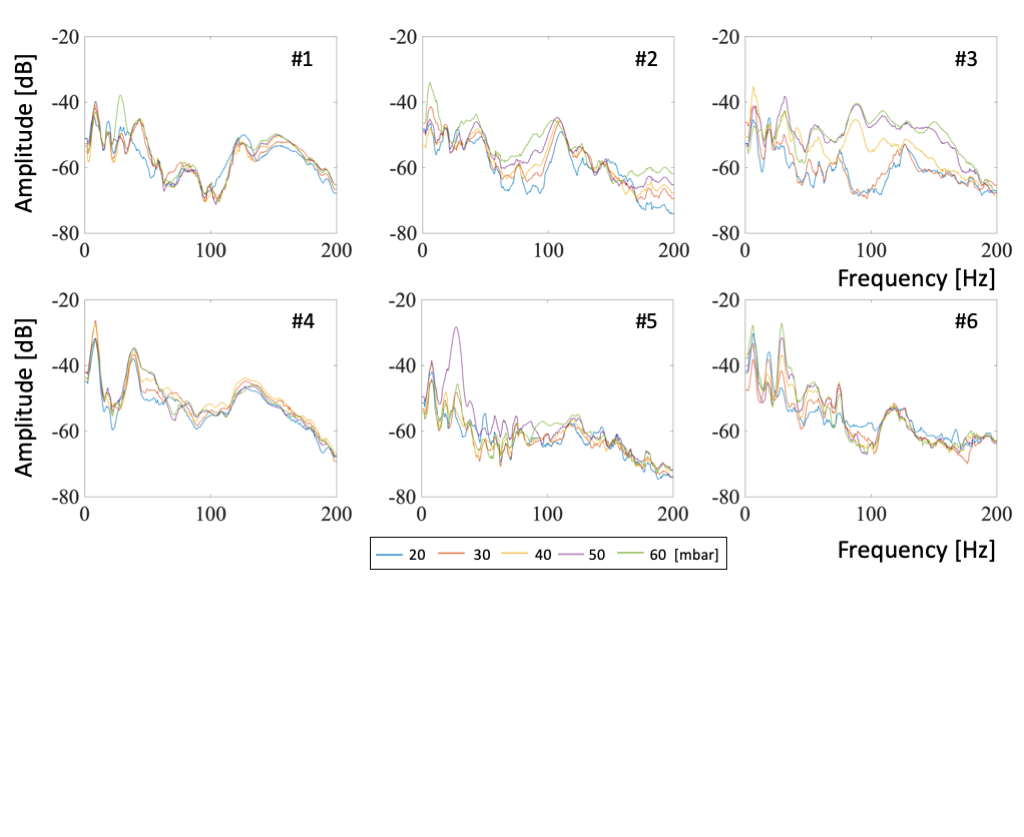


Figure S3 Six power spectra from the eyeball embedded in the orbit with contact tests.


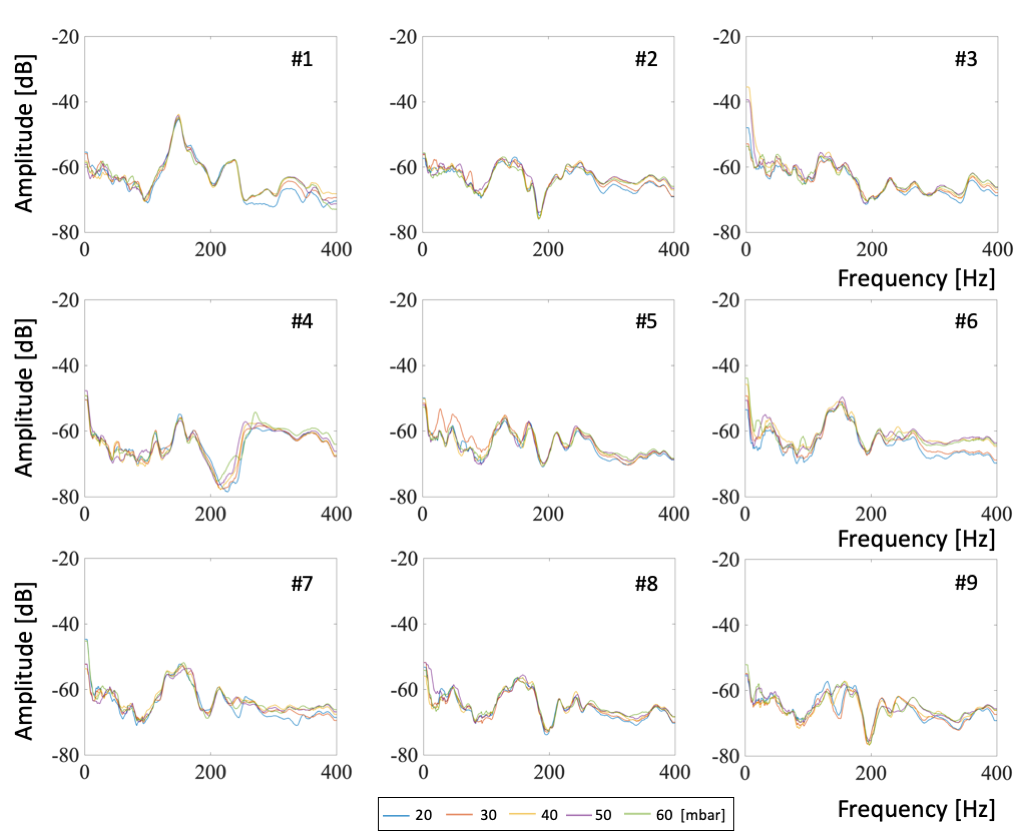


Figure S4 Nine power spectra from the eyeball embedded in the orbit with noncontact tests.


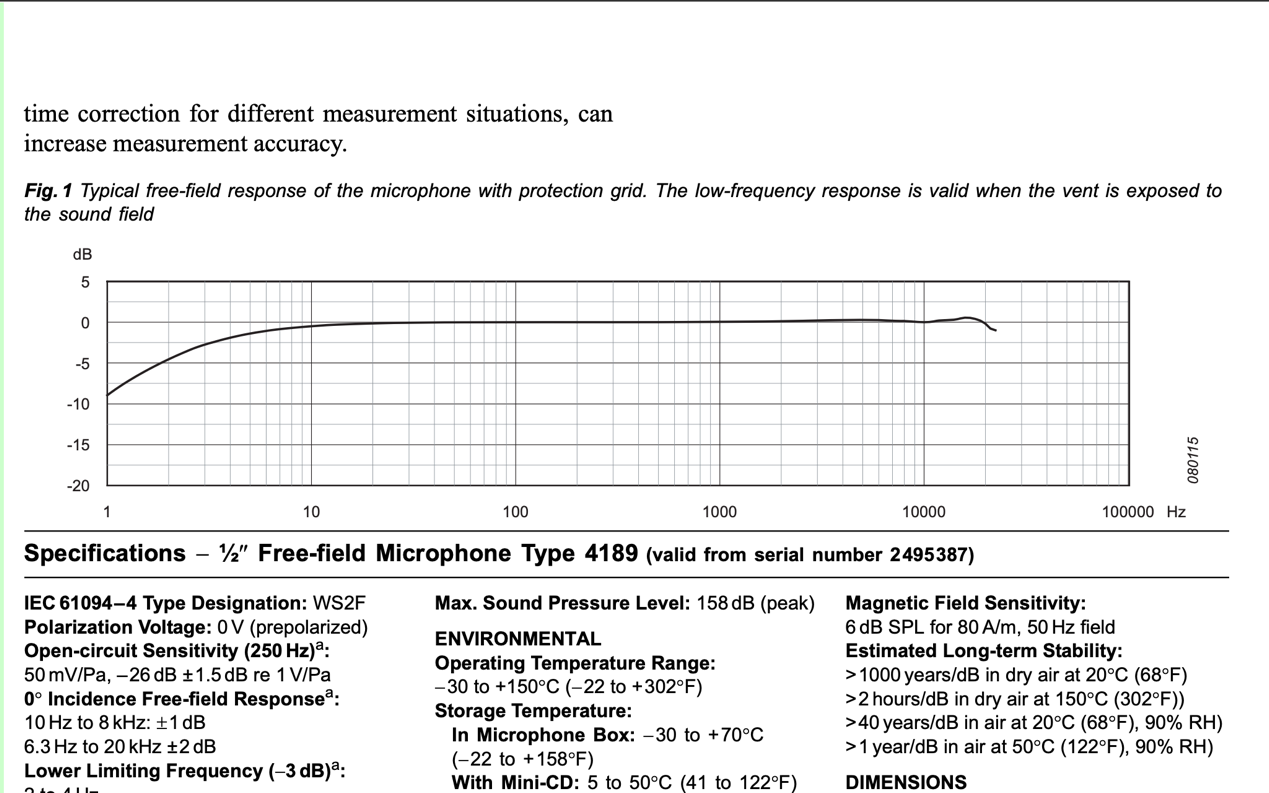


Figure S5 Frequency responses of the acoustic speaker. (a) Free-field response of the microphone with a protection grid (obtained from the specification of type 4189, Brüel & Kjær Vibro). (b) Experimental setup. The microphone contacting (left) and not contacting the speaker with an air gap (right). (c) The frequency response of the speaker, detected by the dish microphone under the contact and non-contact conditions.

Figure S6 Frequency responses from the forehead surface of the skull. (a) Experimental setup: The microphone contacting (upper) and not contacting the forehead surface with a 3-cm air gap (down). (b) The frequency responses of the skull were detected by the dish microphone under the contact and non-contact conditions. Note that the blue curve represents the frequency response of the acoustic source from the speaker, and the results obtained for the non-contact conditions are represented by the ground reference signals in Figure 3.

Figure S7 Frequency responses: (1) The dish microphone contacted the speaker. (2) The dish microphone was placed on the forehead surface. (3) The microphone contacted the eyeball. (4) The dish microphone was in front of the eyeball. (5) The dish microphone was in front of the eyeball.
